# Intranasal administration in modulating depressive-like behavior and reconstructing treatment paradigms through neuroinflammation and neurotrophic pathways

**DOI:** 10.1186/s12951-026-04417-y

**Published:** 2026-04-15

**Authors:** Juan An, Xuejing Li, Zhiqi Li, Jinghui Wang, Yuanming Pan

**Affiliations:** 1 Department of Basic Medical Sciences, Qinghai University Medical College, No.251 of Ningda Road, Xining, 810016 Qinghai China; 2 Cancer Research Center, Beijing Chest Hospital, Capital Medical University, Beijing Tuberculosis and Thoracic Tumor Research Institute, No.9 Beiguan Street, Tongzhou District, Beijing, 101149 China

**Keywords:** Intranasal administration, Depression, Brain-targeted delivery, Nanocarriers, Neuroinflammation, Neuroplasticity

## Abstract

Intranasal administration, as a non-invasive brain-targeted delivery strategy, offers a promising approach for the treatment of central nervous system disorders, particularly depression. Traditional oral or intravenous drug delivery is often limited by the restrictive permeability of the blood-brain barrier and the hepatic first-pass effect, which hinder effective drug accumulation in the brain. In contrast, the intranasal route leverages the olfactory and trigeminal neural pathways to enable direct drug transport from the nasal cavity to the brain, effectively bypassing the blood-brain barrier and significantly enhancing brain drug bioavailability. This article systematically reviews the research progress of intranasal drug delivery in the treatment of depression-like behaviors, highlighting the technological evolution of various drug-loading modalities, including solutions, gels, nanoparticles, in situ gels, and cell membrane biomimetic carriers, and analyzes their differences in nasal mucosal retention time, mucus penetration ability, and brain-targeting efficiency. Furthermore, it elaborates on the multiple antidepressant mechanisms of intranasal materials, such as regulating neurotransmitter systems (e.g., 5-Hydroxytryptamine, Dopamine, Glutamate), inhibiting neuroinflammation (e.g., microglial activation, inflammatory factor release), enhancing neuroplasticity (e.g., promoting hippocampal neurogenesis and synapse formation), and modulating the gut microbiota-gut-brain axis. Simultaneously, the article integrates data from multiple clinical trials, focusing on evaluating the efficacy and safety of intranasal formulations such as esketamine in treatment-resistant depression, highlighting their advantages of rapid onset and high response rates, and discussing management strategies for adverse reactions such as dose individualization, local irritation, and dissociative symptoms. Addressing current technical bottlenecks, such as uneven drug absorption due to nasal physiological differences, challenges in the large-scale production of nanocarriers, and insufficient long-term safety evidence, the article proposes that future research should focus on the development of intelligent responsive nanocarriers, the construction of multimodal synergistic treatment systems, the design of personalized medication regimens guided by precision medicine, and the coordinated advancement of real-world research and regulatory standards. This review aims to provide comprehensive theoretical support and development directions for the clinical translation of intranasal drug delivery in the field of depression treatment.

**Graphical abstract:**

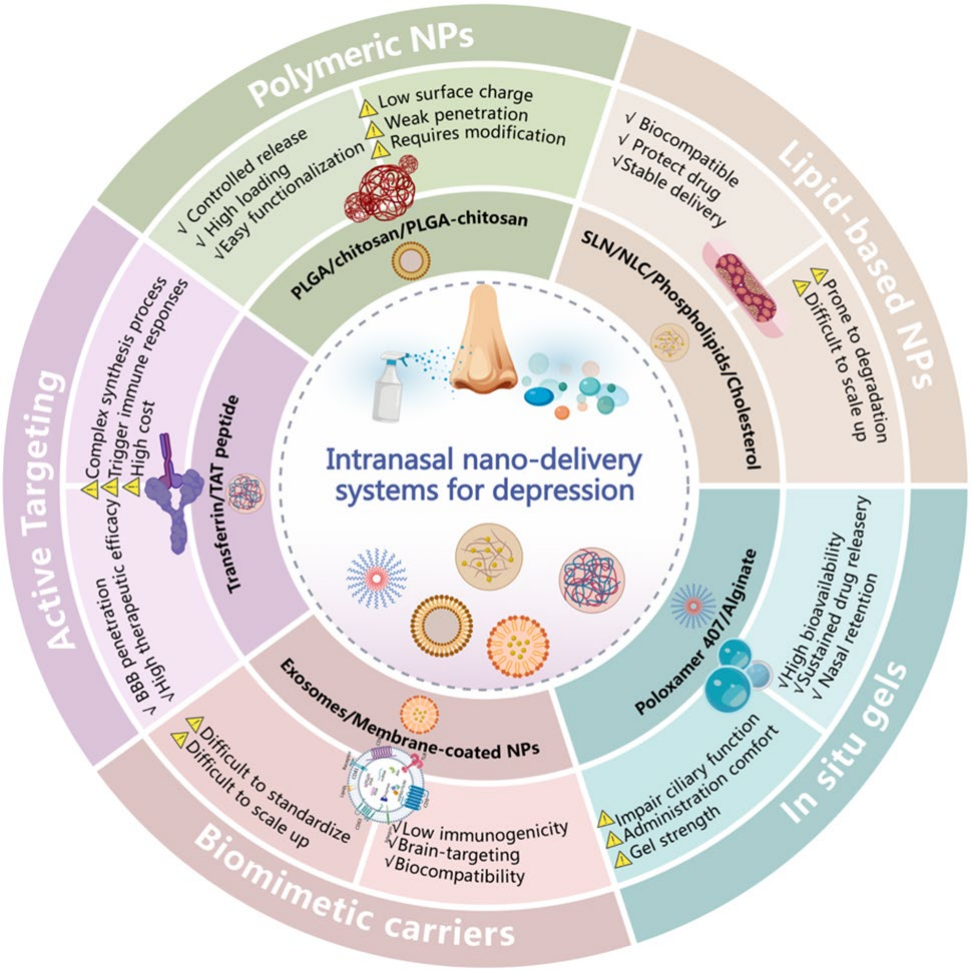

## Introduction

### Research background of intranasal drug delivery

Intranasal administration has emerged as a promising non-invasive strategy for brain-targeted drug delivery, driven by the need to treat central nervous system (CNS) diseases. Conventional routes, such as oral administration or injection, are often ineffective for CNS drugs due to the presence of the blood-brain barrier (BBB), which restricts the entry of most therapeutic agents into the brain [1, 2]. The nasal cavity offers a unique anatomical advantage, as nerves including the olfactory and trigeminal nerves, provide direct pathways to specific brain regions. This enables drugs to bypass the BBB and avoid first-pass metabolism, thereby achieving direct nose-to-brain delivery [1–4] (Table 1).

**Table 1:** Comparison of intranasal, oral, and intravenous administration routes for CNS drug delivery

Parameter	Oral Administration	Intravenous Administration (IV)	Intranasal Administration (IN)	Key Evidence
Brain Targeting Efficiency	Low (< 2% of dose reaches brain) due to BBB and first-pass metabolism [1]	High (100% bioavailability) but non-selective; distributes throughout body [2]	Moderate (5–15% direct nose-to-brain transport); Drug Targeting Efficiency (DTE) values range 150–300% in preclinical studies [5]	Carbamazepine IN showed 107.64% absolute bioavailability vs. IV [6]
Bioavailability	Variable (20–80%) due to hepatic first-pass metabolism and GI degradation [1]	100% absolute bioavailability [2]	Variable (50–170%) depending on formulation; can exceed IV due to bypass of first-pass and direct CNS transport [7]	Sertraline IN showed 166% absolute bioavailability vs. oral in mice [8]
Onset of Action	Slow (weeks for conventional antidepressants; 2–4 weeks typically) [9]	Immediate (seconds to minutes) [2]	Rapid (minutes to hours); 24-h efficacy demonstrated clinically [10]	SHSGS IN showed antidepressant effects within 30 min in mice vs. no effect with oral [10]
Brain Exposure Profile	Fluctuating; peaks and troughs with repeated dosing [1]	Immediate peak followed by rapid decline; requires frequent dosing [2]	Sustained brain levels; prolonged retention in CNS tissues [11]	Sertraline IN provided sustained brain delivery vs. fluctuation with IV/oral [8]
First-Pass Metabolism	Extensive hepatic and intestinal metabolism; reduces active drug reaching brain [1]	Bypasses GI and hepatic first-pass completely[2]	Bypasses GI and hepatic first-pass; direct olfactory/trigeminal pathways to brain [5]	Nasal mucosa avoids GI enzymes and hepatic metabolism [7]
Systemic Side Effects	Common (GI disturbances, weight gain, sexual dysfunction) due to high systemic exposure [9]	Highest risk of systemic toxicity; immediate high concentrations in all organs [2]	Reduced peripheral exposure; lower lung, kidney, and liver accumulation [11]	Zonisamide IN showed lower lung/kidney exposure vs. IV [6]; sertraline IN reduced pulmonary side effects [8]
Patient Compliance	High (convenient, self-administered, suitable for chronic use) [1]	Low (requires healthcare setting, invasive, inconvenient for chronic use) [2]	Moderate (non-invasive, self-administered but requires technique training; in-clinic required for esketamine due to REMS) [12]	Non-invasive and painless, but mucociliary clearance and limited volume per dose constrain delivery [13]
Dosing Limitations	Flexible dosing; wide range of doses possible [1]	Precise dosing; volume and rate controlled [2]	Limited volume per dose (25–200 µL); requires concentrated formulations [14]	Larger particles > 300 nm hinder mucosal transport; mucociliary clearance reduces retention [13]
Barriers to Delivery	Gastric pH, enzymes, intestinal permeability, efflux transporters, BBB [1]	No absorption barriers; but must cross BBB from blood to brain [2]	Nasal mucosal barrier, mucociliary clearance (15–20 min residence time), enzymatic degradation in nasal cavity [15]	Mucociliary clearance and variable nasal anatomy (mucus thickness 10–500 μm) affect absorption [16]
Clinical Evidence in Depression	Extensive; multiple classes approved (SSRIs, SNRIs, TCAs, etc.) [9]	Limited to acute settings (e.g., ketamine infusions in treatment-resistant depression) [15]	Established for esketamine (FDA-approved for TRD); investigational for other antidepressants (sertraline, fluoxetine nanoparticles, SHSGS) [17]	Esketamine IN: Phase III trials show 48% response rate at 4 weeks [18]; real-world studies show 80% response/remission [18–20]

BBB, blood-brain barrier; CNS, central nervous system; DTE, drug targeting efficiency; GI, gastrointestinal; IN, intranasal; IV, intravenous; REMS, Risk Evaluation and Mitigation Strategy; SHSGS, Sihosogansan; SSRI, selective serotonin reuptake inhibitor; SNRI, serotonin-norepinephrine reuptake inhibitor; TCA, tricyclic antidepressant; TRD, treatment-resistant depression

**Fig. 1:**
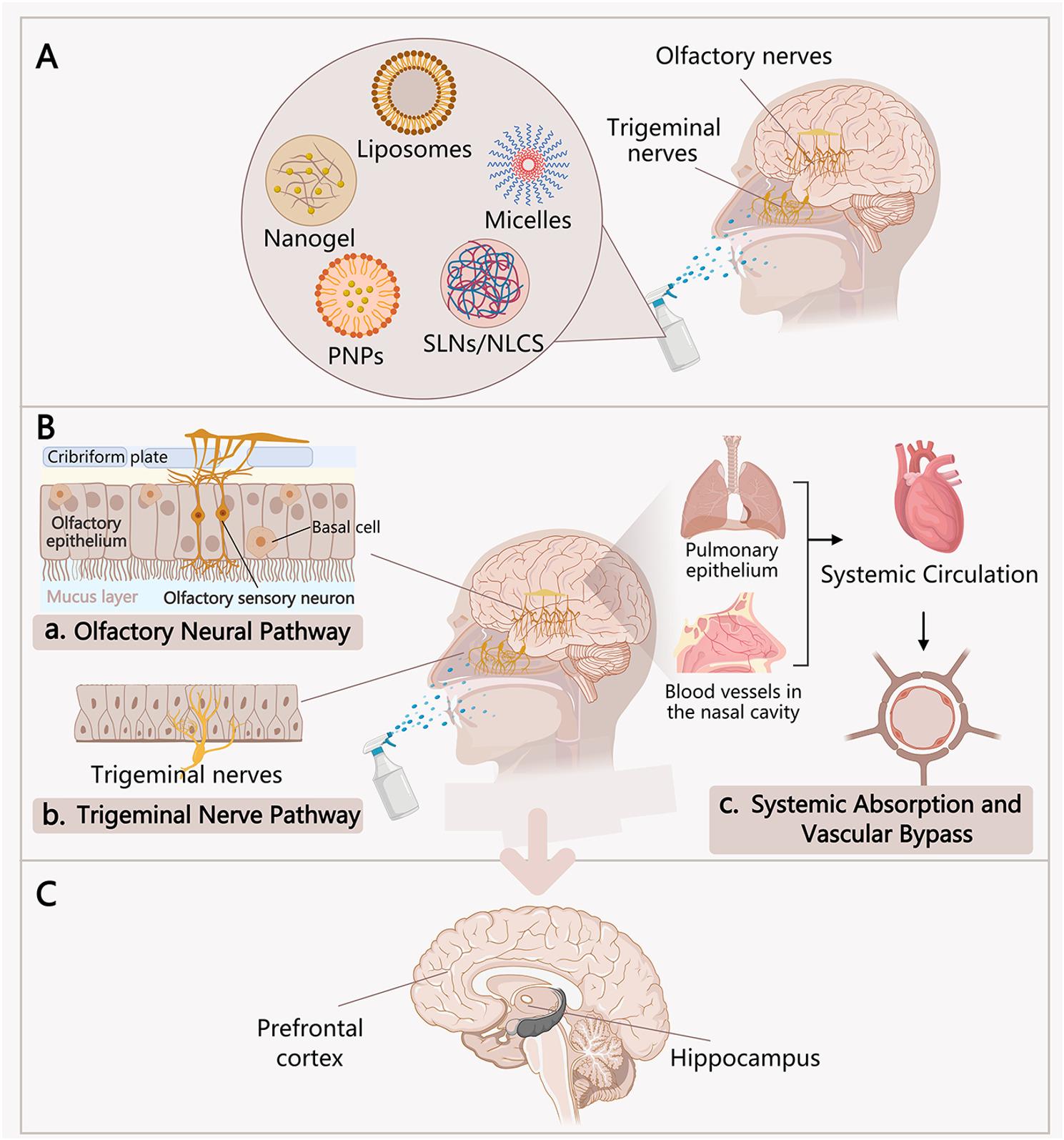
Schematic diagram of delivery system and brain entry routes for intranasal administration. (**A**) Various nanocarriers commonly used for intranasal delivery, such as liposomes, nanogels, and polymeric nanoparticles. (**B**) These carriers can deliver drugs to the central nervous system primarily through three pathways: (a) Olfactory nerve pathway: Carriers cross the olfactory epithelium and are directly transported into the brain via olfactory nerve axons; (b) Trigeminal nerve pathway: Carriers cross the respiratory epithelium, are taken up by trigeminal nerve endings, and are retrogradely transported to the brainstem; (c) Systemic absorption pathway: Carriers are absorbed through the mucosa into the systemic circulation, after which some penetrate the blood-brain barrier to enter the brain. (**C**) The aforementioned pathways ultimately deliver drugs to key brain regions for depression treatment (such as the prefrontal cortex and hippocampus), achieving the goal of targeted therapy by bypassing the blood-brain barrier and increasing drug concentration in the brain

The technological landscape of intranasal delivery systems has evolved substantially to overcome early limitations. Initial formulations, such as simple solutions, were hindered by rapid mucociliary clearance and poor mucosal absorption [4]. In response, advanced carriers have been developed to prolong nasal retention and enhance penetration across the nasal epithelium. The field has progressed from basic solutions to sophisticated nanocarriers, including micelles, liposomes, and more recently to bio-inspired systems that mimic natural cellular properties to evade immune clearance and improve targeting efficiency. that mimic natural cellular properties to evade immune clearance and improve targeting efficiency [3, 4] (Fig. 1).

### Current status and challenges in the treatment of depression

Depression is a leading cause of disability worldwide, yet its treatment continues to face substantial challenges. Current first-line approaches, including antidepressant medication and psychotherapy, are often limited by delayed onset of action, insufficient efficacy, and high rates of relapse. Conventional antidepressants, which primarily modulate monoaminergic neurotransmitters, can take weeks to work and are ineffective for a significant portion of patients, who may develop treatment-resistant depression [21].

The introduction of novel rapid-acting antidepressants, such as esketamine nasal spray, has been considered a breakthrough by demonstrating fast-onset therapeutic effects [17, 22, 23]. Nevertheless, these emerging options are accompanied by their own limitations, including potential adverse effects and unresolved safety concerns associated with long-term use [23]. A fundamental challenge persists in the effective delivery of neuroprotective factors to the brain, as larger therapeutic molecules are routinely excluded by the BBB [1, 24]. Moreover, translating preclinical findings from animal models into effective clinical interventions remains a significant hurdle. While animal models can replicate certain behavioral and neurobiological features of depression, they cannot fully capture the complex emotional and cognitive dimensions of the human condition, thereby complicating the development of universally effective therapies [1–3].

### Purpose and structure of the review

This review aims to systematically elucidate the mechanisms and application prospects of intranasal drug delivery in alleviating depressive-like behaviors. By integrating research progress across dimensions such as drug delivery modes, brain targeting efficiency, neuromodulation mechanisms, behavioral improvements, and safety assessments, it provides theoretical support for the clinical translation of depression treatment. Specific research questions include: (1) the brain targeting efficiency and structure-activity relationships of different intranasal drug delivery modes; (2) the molecular-pathway-behavioral mechanisms by which intranasal drug delivery regulates depressive-like behaviors; (3) the clinical translation potential and safety risks of intranasal drug delivery in depression treatment; (4) translational bottlenecks and solutions between animal models and clinical patients.

The full logical chain follows the research thread of “drug-loading mode → brain-targeting efficiency → neuromodulation mechanism → behavioral improvement → clinical translation barriers → safety closed-loop”:

(1) Drug-Loading Modes for Intranasal Administration: A systematic classification of the physical forms (solutions, gels, nanoparticles, etc.), chemical compositions (liposomes, PLGA, chitosan, etc.), and functional dimensions (passive/active targeting, sustained/rapid release) for intranasal administration was conducted [21]. Key parameters such as nasal mucosal residence time, mucus penetration ability, and brain-targeting efficiency of different drug-loading modes were analyzed [25]. Optimization strategies, such as adding sodium deoxycholate to enhance permeation and conjugating TfR ligands to improve BBB crossing, were proposed in accordance with the requirements of depression-like behavior models [26]. For instance, nanostructured lipid carriers (NLCs) have demonstrated superior brain targeting efficiency with Drug Targeting Efficiency (DTE) values exceeding 200% [26, 27].

(2) Mechanism of action of intranasal materials on depressive-like behaviors: A comprehensive chain from “material entry into the brain → regional distribution → cellular uptake → subcellular localization → signal perturbation → circuit remodeling → behavioral output” is explored. Techniques such as MALDI-IMS spatial metabolomics and CLSM colocalization are combined to reveal the distribution patterns of drugs in the brain [28]. The regulatory effects of intranasal administration on depression-related pathways are elucidated through dynamic monitoring of neurotransmitters (e.g., microdialysis + HPLC detection of 5-HT/DA levels) and assessment of neuroinflammation (e.g., expression of microglial polarization markers Iba1/CD86) [29]. Recent studies demonstrate that intranasal delivery can modulate astrocyte phenotypic transformation, reduce neurotoxic complement C3 release, and activate the Nrf2-Pgc-1α pathway to enhance antioxidant defenses [25].

(3) Evaluation of the Clinical Trial Application of Intranasal Materials for Depression: Focusing on registered/completed clinical trials (such as esketamine nasal spray and BPL-003 trials in ClinicalTrials.gov, NCT05660642, NCT05870540), analyze the quality of trial design (randomized double-blind, sample size calculation), efficacy data (effect size, NNT), and safety endpoints (local nasal reactions, central adverse events), critically discussing the limitations of extrapolating animal data to clinical settings. Recent Phase 2a data for BPL-003 (intranasal 5-MeO-DMT) showed that 55% of treatment-resistant depression patients achieved clinical response on the day after dosing, with 45% in remission at week 12. A Phase 2b trial with 196 patients has completed enrollment, with results expected in mid-2025.

(4) Safety Evaluation: Encompassing biocompatibility tests required by the ISO 10,993 standard, including local safety (nasal mucosal histopathology, olfactory electrogram), systemic toxicity (RES organ accumulation, immunogenicity), and long-term safety (NHP repeated-dose studies), establishing a predictive model of “material characteristics-biological interface reactions-chronic pathological outcomes” [25, 27, 30].

This review integrates multidisciplinary research advances to provide a comprehensive perspective on the application of intranasal drug delivery in the treatment of depressive-like behaviors, while highlighting current research gaps and future directions, thereby contributing to technological innovation and clinical translation in depression therapy.

### Methodology

This review was conducted and reported in accordance with the Preferred Reporting Items for Systematic Reviews and Meta-Analyses (PRISMA) 2020 statement and guidelines for scoping reviews to ensure transparency and reproducibility.

#### Search strategy

A systematic literature search was performed in March 2026 across the following electronic databases: PubMed/MEDLINE, Web of Science Core Collection, Scopus, Embase, Cochrane Library, and Google Scholar (first 200 records). The search timeframe covered each database from its inception to March 2026. The search strategy combined Medical Subject Headings (MeSH) terms and free-text keywords, including: “intranasal administration,” “nose-to-brain delivery,” “nanoparticles,” “nanocarriers,” “depression,” “major depressive disorder,” “antidepressants,” and “blood-brain barrier.” The complete search strategy for PubMed is provided in Supplementary Table 1. Additionally, reference lists of included studies were manually searched (snowball searching), and relevant reviews and conference abstracts were screened to identify potentially eligible studies.

#### Inclusion and exclusion criteria

Inclusion criteria were defined according to the PICO (Population, Intervention, Comparison, Outcome) framework: (1) Population: Preclinical studies (animal models of depression) and clinical studies (patients with depression), with no restrictions on species, age, sex, or ethnicity. (2) Intervention: Nanoformulations administered via the intranasal route, including but not limited to polymeric nanoparticles, solid lipid nanoparticles, nanostructured lipid carriers, nanoemulsions, liposomes, and in situ gelling systems. (3) Comparison: Non-nanoformulations (e.g., free drug solutions), conventional administration routes (e.g., oral, intravenous), or placebo/blank controls. (4) Outcome: Pharmacokinetic parameters (brain targeting efficiency, bioavailability), pharmacodynamic outcomes (behavioral test results), mechanistic findings (neurotransmitter levels, neuroinflammatory markers, neurotrophic factor expression), and safety evaluations (local tolerability, cytotoxicity, biodistribution).

Exclusion criteria included: (1) non-English publications; (2) conference abstracts, reviews, editorials, commentaries, case reports, and other non-original research; (3) studies reporting only in vitro experiments without in vivo validation; (4) studies investigating intranasal delivery without nanoformulations; (5) articles with unavailable full text.

#### Study selection and data extraction

All retrieved records were imported into EndNote X20, and duplicates were removed. Two reviewers independently performed study selection: initial screening based on titles and abstracts to exclude clearly irrelevant studies, followed by full-text review of potentially eligible articles with reasons for exclusion documented. Disagreements were resolved through discussion or consultation with a third reviewer. The selection process was documented using a PRISMA flow diagram.

Data extraction was performed independently by two reviewers using a standardized form. Extracted information included: first author and year of publication, country, nano-formulation type and physicochemical properties (particle size, zeta potential, encapsulation efficiency, drug loading), animal model/patient characteristics, administration regimen (dose, frequency), primary outcome measures, mechanistic findings, and safety data. Corresponding authors were contacted for missing data when necessary.

#### Data synthesis and analysis

Due to anticipated heterogeneity in interventions, study populations, and outcome measures, meta-analysis was not performed. A narrative synthesis approach was adopted, organizing results according to the following themes: (1) types and characteristics of intranasal nano-formulations; (2) intervention targets related to depression pathophysiology (neurotransmission, neuroinflammation, neuroplasticity, gut-brain axis, epigenetics); (3) brain targeting efficiency and pharmacokinetic profiles; (4) safety assessments and challenges for clinical translation.

#### Quality assessment and risk of bias

The SYRCLE (Systematic Review Centre for Laboratory animal Experimentation) Risk of Bias tool was used to assess methodological quality of preclinical studies. Clinical studies were evaluated using the Cochrane Risk of Bias tool (RoB 2.0). Quality assessment was conducted independently by two reviewers, with discrepancies resolved through discussion. Studies were not excluded based on quality scores, but methodological quality was considered in result interpretation.

## Formulation strategies for nose-to-brain delivery in depression treatment

**Fig. 2:**
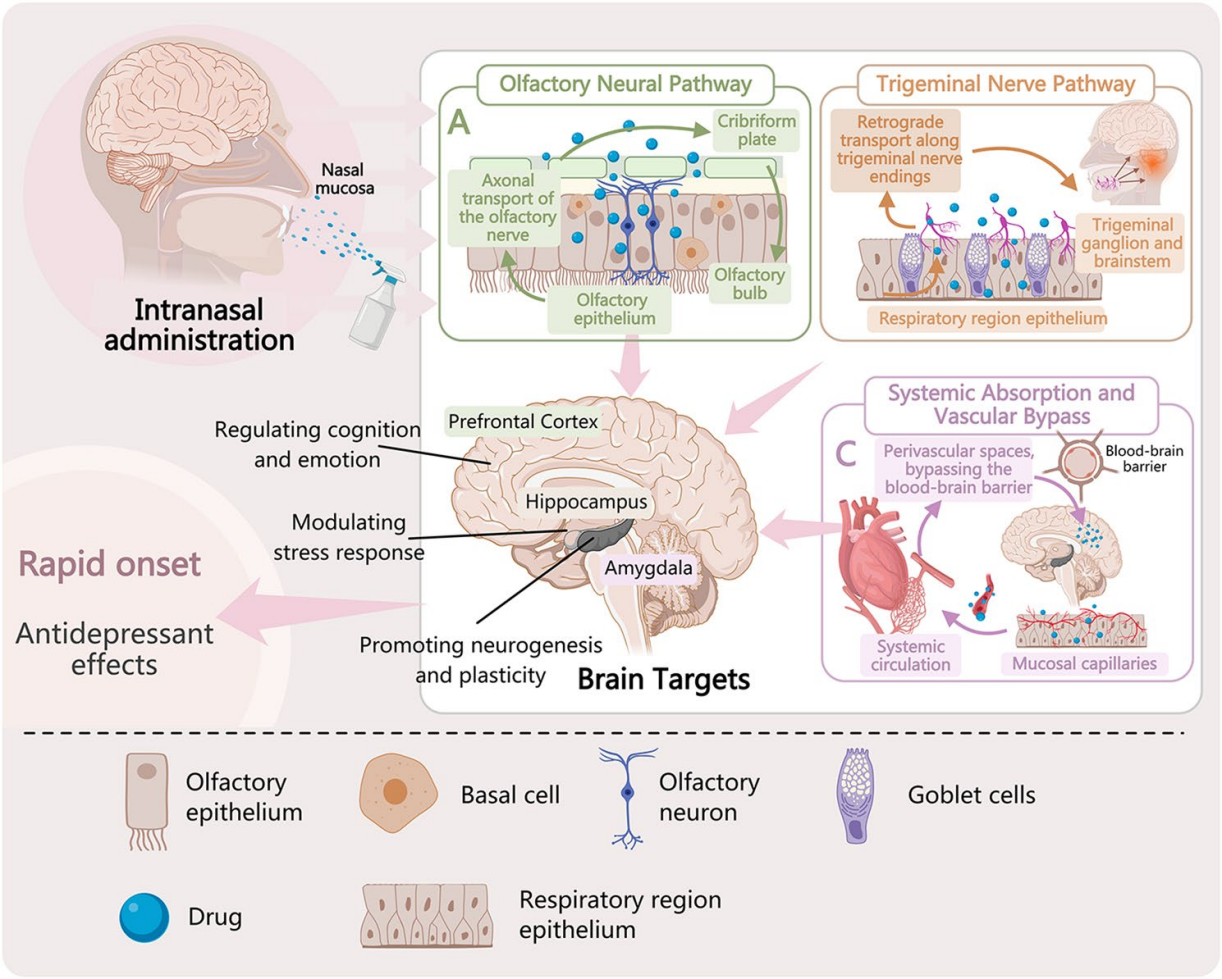
Schematic diagram of brain-targeted delivery pathways via intranasal administration and associated antidepressant effects. (**A**) Various nanocarriers commonly used for intranasal delivery, such as liposomes, nanogels, and polymeric nanoparticles., (**B**) These carriers can deliver drugs to the central nervous system primarily through three pathways:, (a) Olfactory nerve pathway: carriers cross the olfactory epithelium and are directly transported into the brain via olfactory nerve axons;, (b) Trigeminal nerve pathway: carriers cross the respiratory epithelium, are taken up by trigeminal nerve endings, and are retrogradely transported to the brainstem;, (c) Systemic absorption pathway: carriers are absorbed through the mucosa into the systemic circulation, after which some penetrate the blood-brain barrier to enter the brain., (**C**) The aforementioned pathways ultimately deliver drugs to key brain regions for depression treatment, (such as the prefrontal cortex and hippocampus), achieving the goal of targeted therapy by bypassing the blood-brain barrier and increasing drug concentration in the brain

Intranasal drug delivery systems are designed to overcome three primary barriers: rapid mucociliary clearance, enzymatic degradation in the nasal cavity, and inefficient transport across the olfactory epithelium [2, 31–33] (Fig. 1A and B). The evolution of these formulations reflects a shift from simply prolonging nasal retention to actively facilitating brain targeting (Fig. 1C). This section outlines the key design principles-delivery modes, material properties, and therapeutic applications-that guide current research in treating depression-like behaviors [2, 4, 34] (Fig. 2).

### Design principles of drug delivery modes

Nasal drug delivery systems can be categorized by their physical form, chemical composition, and functional mechanism [4]. Rather than detailing every variant, the following principles summarize how these systems are engineered to meet the specific demands of depression intervention, such as rapid onset or sustained synaptic targeting.

Physical Form and Retention. The residence time of a formulation in the nasal cavity is a fundamental determinant of drug absorption. Early solutions are cleared within minutes, resulting in low bioavailability [35]. To address this, systems are designed to increase viscosity or form gels upon contact with nasal mucosa [34]. Gels and in situ gelling systems (e.g., temperature-responsive polymers) can extend retention to several hours by resisting mucociliary clearance. Microparticles (1–10 μm) enhance retention through mechanical entrapment, while nanoparticles (typically < 200 nm) are favored for their ability to penetrate the mucus layer and access the olfactory epithelium, facilitating direct nose-to-brain transport [36] (Fig. 1A).

Chemical Composition and Carrier Function. The choice of carrier material determines drug protection, release profile, and interaction with biological surfaces [37, 38]. Lipid-based carriers (e.g., liposomes, solid lipid nanoparticles) offer high biocompatibility and are well-suited for lipophilic antidepressants, though they may require stabilization against nasal enzymes [4], Synthetic polymers like PLGA provide controlled, sustained release over days, making them ideal for long-acting formulations [39]. Natural polysaccharides, particularly chitosan, are valued for their mucoadhesive properties; their positive charge enables electrostatic binding to the negatively charged nasal mucosa, prolonging contact time [40, 41]. Emerging biomimetic carriers, such as exosomes, leverage native cell-like surfaces to evade immune recognition and enhance brain distribution efficiency [40–46] (Fig. 1A).

Functional Targeting and Release. Delivery modes are further classified by their mechanism of action. Passive targeting relies on optimized physicochemical properties (size, charge) to achieve brain distribution [47, 48]. Active targeting involves surface modification with ligands (e.g., transferrin, antibodies) that bind to receptors on nasal epithelial cells or neurons, significantly improving brain uptake [29]. Release kinetics can be tailored for specific therapeutic needs: rapid-release systems (e.g., micelles) provide fast drug availability for acute intervention [49, 50], while sustained-release systems (e.g., PLGA microspheres) maintain therapeutic levels over extended periods [51–53]. Multi-drug co-delivery platforms are also being developed to achieve synergistic effects, such as combining serotonergic modulation with anti-inflammatory action.

### Material properties and biological performance

The efficacy and safety of intranasal formulations are directly linked to the physicochemical properties of their constituent materials. These properties govern not only drug loading and release but also interactions with nasal tissue and, ultimately, brain targeting [53] (Fig. 2).

Mucoadhesion and Permeation. Materials with strong mucoadhesive properties, such as cationic chitosan, bind to mucosal surfaces via electrostatic interactions, prolonging retention [54, 55]. This adhesion can also transiently open tight junctions between epithelial cells, enhancing paracellular drug transport [55–57]. However, high concentrations of such materials may induce local inflammatory responses, necessitating a balance between efficacy and tolerability [58]. Studies have shown that chitosan-based formulations can significantly improve drug bioavailability by 31- to 42-fold compared to simple solutions [59].

Particle Size and Distribution. Nanoparticle size is a critical determinant of transport pathway [53]. Particles smaller than 100 nm are more efficiently transported via olfactory neurons directly to the brain [56, 58]. Particles between 100 and 200 nm predominantly traverse the mucus layer and may be taken up by epithelial cells, while larger particles (> 200 nm) are largely retained on the mucosal surface [58, 60, 61]. A narrow size distribution (low polydispersity index) ensures predictable and consistent in vivo behavior [53, 56]. Research indicates that nanoparticles of approximately 100 nm have longer retention duration in nostrils and slower mucociliary clearance than larger ones [60–62].

Biocompatibility and Regulatory Status. Materials approved by regulatory agencies (e.g., PLGA, poloxamers) offer established safety profiles and are preferred for clinical translation [4, 64]. Natural polymers generally exhibit low toxicity but may have limitations in drug loading or stability [65]. Synthetic materials provide tunable degradation rates but may require surface modification (e.g., PEGylation) to reduce immune recognition [64]. Neurotoxicity is a critical consideration; cationic polymers with high charge density can cause mitochondrial damage, whereas materials like chitosan have high lethal dose thresholds, indicating a wider safety margin [66–68] (Table 2).

**Table 2:** Intranasal antidepressant nanocarrier drug delivery systems: Models, characteristics, and representative studies

Drug delivery system	Core materials/composition	Main advantages	Limitations	Representation applications and effects in depression models
Polymer nanoparticles	PLGA, chitosan, PLGA-chitosan composites	Controllable degradation, high drug loading capacity, and can achieve sustained release or targeting through modifications	Surface charge is usually low, and mucosal penetration ability is weak, which may require modification for improvement	Loading dextromethorphan PLGA-chitosan NP: brain concentration is 5 times higher than the oral group, sucrose preference in CUMS rats increased by 30%, and immobility time in FST decreased by 40% [69].
Lipid-based nanocarriers	phospholipids, cholesterol (liposomes); solid/liquid lipids (SLN/NLC)	High biocompatibility and are easy to encapsulate lipophilic drugs, with better drug loading and stability for NLC	Liposomes are easily degraded by nasal enzymes; challenging for large-scale production	Fluoxetine-loaded NLC: brain bioavailability increased 3 times compared to the oral group, upregulating hippocampal BDNF expression [65].
In situ gel systems	Poloxamer 407 (thermosensitive), alginate (ion-sensitive)	Significantly extend the nasal retention time, achieving sustained drug release and increasing bioavailability	The gel strength needs to be balanced with administration comfort; it may affect ciliary function	Icaritin-loaded nanogel-thermosensitive gel: 30 min intracerebral distribution, CUMS rats’ sucrose preference ↑40%, plasma IL-6 ↓50% [70].
Bionic carriers	Exosomes (such as those derived from Chlorella)	Cell membrane encapsulated nanoparticles, exhibit extremely high biocompatibility and brain targeting ability, low immunogenicity, and can overcome multiple barriers	Extraction, drug loading, and large-scale production are challenging, and standardization is difficult	Exosomes derived from Chlorella: after nasal administration, they improve behavioral phenotype in LPS and CUMS depression models, and their brain targeting efficiency is significantly higher than that of synthetic nanoparticles, accompanied by downregulation of neuroinflammation (TNF-α) in the hippocampus [25].
Active targeting modification system	Nano-carriers connect with targeted ligands like transferrin and TAT peptides	It actively enhanced blood-brain barrier penetration and accumulation in brain lesions, improving efficacy and reducing systemic exposure	The synthesis process is complex, and ligands may trigger immune responses, resulting in high costs	Transferrin-modified risperidone NP: brain targeting efficiency (DTE%) reaches 92%, with the onset time in the social defeat model shortened by 2 h compared to the intravenous group [71].

### Translational applications in depression-like behavior models

The therapeutic potential of intranasal delivery systems is validated through preclinical studies using animal models of depression, particularly the chronic unpredictable mild stress (CUMS) model [63]. These studies consistently demonstrate that optimized formulations outperform traditional routes (oral, intravenous) in terms of brain bioavailability, onset of action, and behavioral outcomes [21, 64] (Table 1).

Enhanced Brain Targeting. Across multiple studies, intranasal administration of antidepressant-loaded nanoparticles (e.g., PLGA-chitosan, nanostructured lipid carriers) results in brain concentrations 3- to 5-fold higher than oral delivery [64]. For example, venlafaxine-loaded PLGA nanoparticles (190–210 nm) administered intranasally for just 7 days reversed the depressive-like phenotype, showing significant antidepressant effects compared to free venlafaxine [64]. Active targeting via surface modification strategies further increases brain targeting efficiency. Studies on biomineralized silk fibroin nanoparticles demonstrated reliable brain targeting with minimal systemic distribution via intranasal administration, enabling efficient delivery along the nasal cavity-olfactory bulb-brain axis [21, 29]. This enhanced delivery translates directly to improved efficacy in behavioral tests, such as increased sucrose preference (indicating reduced anhedonia) and decreased immobility in forced swim and tail supspension tests [10, 64].

Rapid and Sustained Effects. The onset of antidepressant action can be significantly shortened with intranasal delivery. Research on intranasal sihosogansan demonstrated rapid anxiolytic and antidepressant effects at 30 min post-administration in mice, whereas oral administration had no significant effect at the same time point [8]. Venlafaxine-loaded PLGA nanoparticles administered intranasally provided faster antidepressant-like effects in animal models compared to conventional formulations [64]. Conversely, sustained-release formulations maintain elevated brain drug levels for extended periods, reducing dosing frequency and providing prolonged behavioral improvement. Curcumin-loaded thermosensitive gels and nanostructured lipid carriers provide sustained release profiles, maintaining therapeutic concentrations and showing superior antidepressant activity [29, 65]. This flexibility allows formulation design to match the specific clinical requirement, whether for acute crisis intervention or maintenance therapy.

Mechanistic Advantages. Beyond pharmacokinetic benefits, intranasal delivery can enhance pharmacodynamic outcomes. For example, carriers that facilitate brain delivery of neurotrophic factors promote hippocampal neurogenesis in stressed animals [8, 29, 65]. Intranasal delivery of biomineralized silk fibroin nanoparticles encapsulating tetrahydroxystilbene glucoside and berberine significantly improved behavioral outcomes (*p* < 0.0001) and enhanced BDNF expression with ∼6.2-fold higher than that of the MDD group, achieved through synergistic effects of M2-like microglial polarization, microglial restoration, and neuroinflammatory elimination in the hippocampus region [14]. Studies on AC5216 nanoemulsions showed significant reduction in microglial activation and amelioration of neuroinflammation [63]. Co-delivery systems combining antidepressants with functional excipients address multiple pathological pathways in depression, resulting in synergistic therapeutic effects. Fluoxetine-loaded nanostructured lipid carriers incorporating saffron oil demonstrated synergistic antidepressant effects through enhanced permeation and combined pharmacological activities, with ex vivo permeation studies on goat nasal mucosa revealing significantly enhanced nasal mucosal permeability [64]. These findings underscore that intranasal formulation is not merely a delivery method but an integral component of the therapeutic strategy [21].

In summary, the rational design of intranasal delivery systems-integrating physical form, material properties, and functional targeting-enables precise, efficient, and safe intervention in depression-like behaviors [21, 63]. The field is moving toward increasingly sophisticated carriers that not only overcome biological barriers but also actively participate in the therapeutic response [29].

## Mechanisms of intranasal delivery in treating depression-like behaviors

**Fig. 3:**
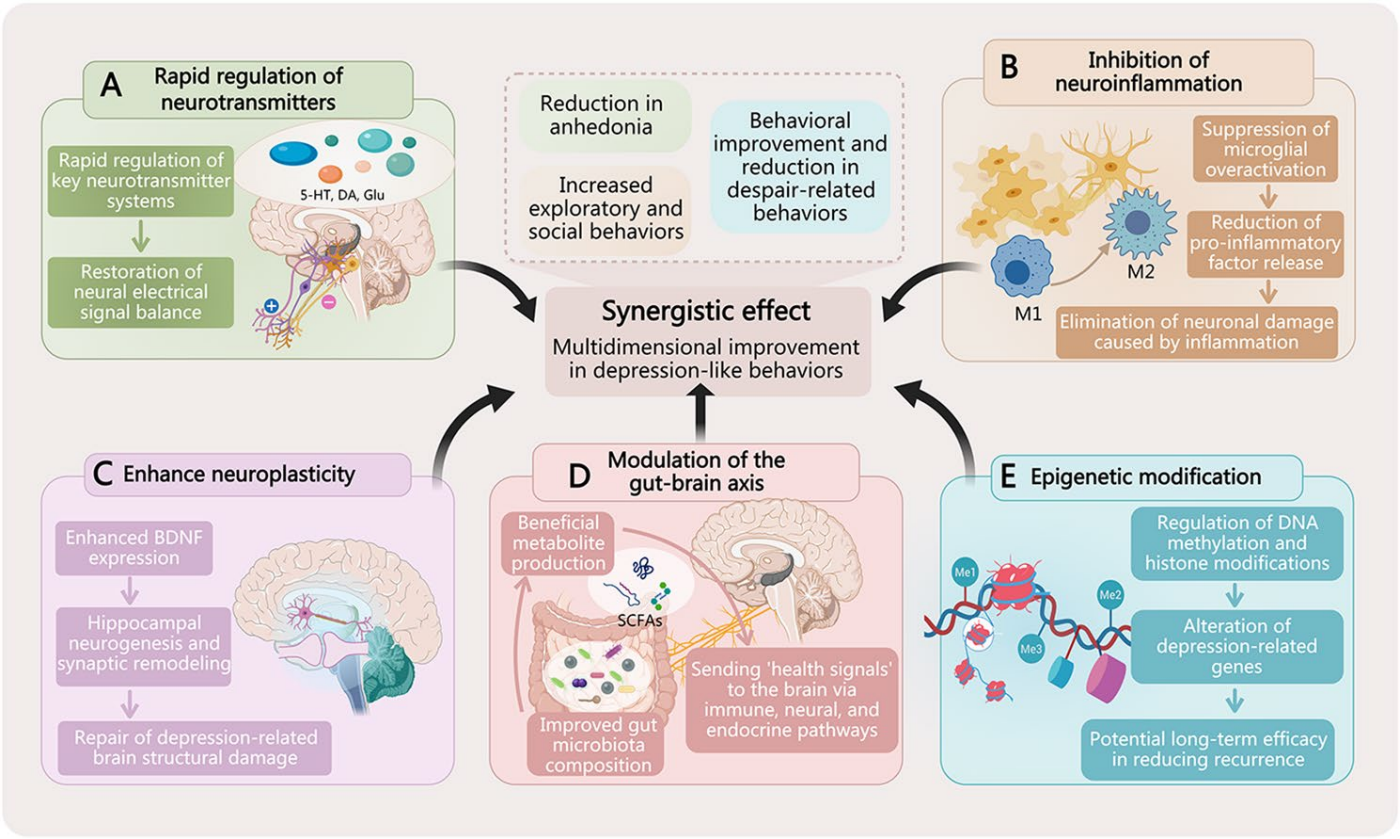
Schematic summary of multitarget and synergistic mechanisms underpinning the antidepressant effects of intranasal drug delivery. (**A**) Rapid neurotransmitter regulation: Intranasal delivery facilitates fast modulation of key neurotransmitter systems (e.g., serotonin [5-HT], dopamine [DA], glutamate [Glu]), helping to restore neural signal balance. (**B**) Inhibition of neuroinflammation: The therapy suppresses the overactivation of pro-inflammatory microglia (M1 phenotype) and promotes their transition to an anti-inflammatory state (M2 phenotype), reducing neuroinflammation. (**C**) Enhancement of neuroplasticity: Interventions upregulate brain-derived neurotrophic factor (BDNF) expression, promoting hippocampal neurogenesis and synaptic remodeling to repair depression-related structural damage. (**D**) Modulation of the gut-brain axis: Treatment improves gut microbiota composition, leading to the production of beneficial metabolites (e.g., short-chain fatty acids). These metabolites subsequently send regulatory signals to the brain via immune, neural, and endocrine pathways. (**E**) Epigenetic modification: The approach induces changes in epigenetic markers, such as DNA methylation and histone modifications, which can alter the expression of depression-related genes, contributing to potential long-term efficacy

Intranasal drug delivery systems exert their antidepressant effects through multiple, interconnected mechanisms. Rather than operating in isolation, these mechanisms form an integrated network that restores central nervous system function. This section synthesizes the primary pathways, enhanced brain delivery, neurotransmitter modulation, neuroinflammation regulation, neuroplasticity promotion, neural circuit modulation, gut-brain axis interactions, and epigenetic regulation and examines how they collectively contribute to improved behavioral outcomes (Fig. 3).

### Integrated mechanistic framework

The therapeutic efficacy of intranasal formulations arises from a cascade of events that begin with efficient brain targeting and culminate in behavioral improvement. These mechanisms operate on different timescales and across multiple levels of biological organization, from molecular to circuit-level changes.

Brain Delivery as the Enabling Mechanism. The fundamental advantage of intranasal administration is its ability to bypass the blood-brain barrier through anatomical connections between the nasal cavity and the central nervous system [1, 66]. Two primary pathways mediate this delivery:

The olfactory pathway involves transport from the olfactory epithelium to the olfactory bulb via neuronal or paracellular routes [67, 68]. Drugs and nanocarriers can enter olfactory neurons through endocytosis and undergo retrograde axonal transport or diffuse through intercellular spaces to reach the brain parenchyma [53]. Carrier properties critically influence delivery efficiency: nanoparticles in the 10–100 nm range penetrate the olfactory epithelium most effectively, while surface modification with targeting ligands (e.g., transferrin receptor antibodies) enhances brain-specific accumulation [54, 72] (Fig. 2).

The trigeminal pathway delivers drugs from the nasal respiratory epithelium to the brainstem and broader brain regions [1, 67]. Sensory nerve endings in the nasal mucosa internalize carriers and transport them retrogradely to the trigeminal ganglion, from which they diffuse to various brain areas [68]. Perivascular transport along blood vessels provides an additional route, particularly for larger molecules [53, 66]. Research has confirmed that intranasally-administered tracers rapidly reach the trigeminal nerve with gradual increase in fluorescence signal at the perineural space over time [68] (Fig. 2).

Mucociliary clearance represents the primary barrier to efficient delivery [34]. Formulation strategies to overcome this include mucoadhesive carriers (e.g., chitosan-based systems) that bind to mucosal surfaces via electrostatic interactions, mucus-penetrating nanoparticles (e.g., PEGylated systems) that avoid entrapment in the mucus network, and in situ gelling systems that prolong nasal retention [34, 73]. Molecular dynamics simulations have revealed that mucoadhesive materials like hydroxypropyltrimethyl ammonium chloride chitosan (HTCC) have strong interactions with mucin MUC5AC, while mucus-penetrating materials like PEG-PLGA show minimal interaction, providing mechanistic insights for rational carrier design [73]. Quantitative assessment techniques—radionuclide tracing, mass spectrometry imaging, gamma scintigraphy, and confocal microscopy—have confirmed that optimized formulations achieve significantly higher brain concentrations than conventional administration routes [72, 74].

Convergent Mechanistic Pathways. Once delivered to the brain, intranasal materials engage multiple therapeutic mechanisms that converge on the restoration of normal neural function:

Neurotransmitter systems are directly modulated by delivered antidepressants. Intranasal administration of SSRIs (e.g., sertraline) rapidly elevates synaptic serotonin levels in the prefrontal cortex, with studies demonstrating that intranasal sertraline significantly reduces adrenaline while increasing serotonin and melatonin following administration [8]. Dopamine and norepinephrine systems are similarly enhanced by agents like desvenlafaxine. Beyond monoamines, glutamatergic and GABAergic systems represent critical targets. Ketamine’s rapid antidepressant effect involves NMDA receptor antagonism, leading to increased glutamate release and subsequent synaptic strengthening [75]. Research has shown that intranasal ketamine shifts the cortical excitation/inhibition balance in favor of excitation in the medial prefrontal cortex, with a greater magnitude of effect compared to intraperitoneal administration [76]. Ginkgolide B and geniposide enhance GABAergic transmission, providing anxiolytic effects. The efficiency of intranasal delivery accelerates the onset of these neurotransmitter changes compared to oral administration (Fig. 3A).

Neuroinflammation is a key pathological driver in depression. Chronic stress activates microglia, which release pro-inflammatory cytokines (IL-1β, IL-6, TNF-α) and activate inflammasome complexes (e.g., NLRP3), contributing to neuronal dysfunction. Intranasal delivery enables efficient brain targeting of anti-inflammatory agents. Berberine inhibits hippocampal IL-6 and TNF-α release by suppressing NF-κB activation. Lithium salts reduce NLRP3 inflammasome activation and lower IL-1β and IL-18 levels. Low-dose lipopolysaccharide paradoxically exerts antidepressant effects by promoting M2 microglial polarization and anti-inflammatory cytokine release (IL-10). Resveratrol and curcumin, when delivered intranasally, achieve sufficient brain concentrations to suppress microglial activation and oxidative stress, effects difficult to achieve with systemic administration [77] (Fig. 3B).

Neuroplasticity enhancement represents a fundamental mechanism for sustained recovery. Brain-derived neurotrophic factor (BDNF) is central to this process, supporting neuronal survival, synaptic growth, and hippocampal neurogenesis. Intranasal delivery overcomes the blood-brain barrier that normally prevents protein-based therapeutics from reaching the brain. BDNF-HA2TAT/AAV, a fusion construct with cell-penetrating peptides, achieves significant hippocampal enrichment after intranasal administration, increasing BDNF levels, promoting neurogenesis in the dentate gyrus, and enhancing synaptic protein expression (synaptophysin, PSD-95) [78]. Research has confirmed that intranasal infusion of GALR2 and Y1R agonists promotes neuroblasts proliferation in the dentate gyrus and induces BDNF through increased formation of Y1R-GALR2 heteroreceptor complexes [79]. Intranasal oxytocin has also been shown to restore decreased BDNF expression in the hippocampus [77]. Ketamine activates mTOR signaling, rapidly increasing synaptic density and dendritic spine maturation [75, 76]. These structural changes correlate with sustained behavioral improvements, distinguishing neuroplasticity enhancement from transient symptomatic relief (Fig. 3C).

Neural circuit modulation occurs at the systems level. Depression involves disrupted functional connectivity between brain regions, particularly within fronto-limbic circuits. Intranasal materials normalize these network abnormalities: ketamine alters default mode network connectivity, with studies demonstrating that intranasal (R)-ketamine modulates specific human brain networks by attenuating long-range synchrony in the supplementary motor area/middle cingulate cortex, an effect spatially correlated with serotonin, norepinephrine, and acetylcholine neurotransmitter profiles [80]; Oxytocin reduces amygdala hyper-reactivity, with fMRI investigations showing enhanced connectivity between amygdala and bilateral insula and middle cingulate gyrus after intranasal oxytocin administration [81]. and BDNF delivery enhances hippocampal-prefrontal coupling. EEG studies have further revealed that responders to intranasal ketamine and esketamine show increased functional connectivity and decreased entropy in the frontal region compared to non-responders [82]. These circuit-level effects are likely the substrate for comprehensive behavioral recovery.

Gut-brain axis interactions represent an emerging mechanism. Intranasal materials can modulate the gut microbiota composition and metabolism through pathways not yet fully elucidated. While direct evidence for chlorella-derived extracellular vesicles remains limited, research has established that extracellular vesicles (exosomes) function as key inflammatory mediators that can translocate across epithelial barriers and trigger local and central inflammation [83]. The nasal microbiome itself is increasingly recognized as a potential modulator of neuroinflammation in major depressive disorder, with dysbiosis linked to transepithelial translocation of microorganisms and their metabolites, disrupting epithelial barriers and favoring vascular permeability [80]. Studies have demonstrated that nasal Staphylococcus aureus carriage promotes depressive behavior in mice via degradation of sex hormones (estradiol and testosterone), leading to decreased levels of dopamine and serotonin in the brain, providing direct evidence for a nose-brain axis mediated by microbial metabolism [84]. These inflammatory and metabolic changes correlate with reduced neuroinflammation and improved depressive-like behaviors, suggesting a gut-brain axis mediated by immune and metabolic signaling that may be influenced by intranasal interventions (Fig. 3D).

Epigenetic regulation provides a molecular basis for sustained gene expression changes. Studies have demonstrated that ketamine produces antidepressant-like effects through phosphorylation-dependent nuclear export of histone deacetylase 5 (HDAC5) in rats [85, 86]. This mechanism inhibits histone deacetylases, increasing histone acetylation and promoting transcription of plasticity-related genes. Research has also shown that intranasal treatment with lixisenatide, a GLP-1 analog, increases phosphorylation of CREB protein in hippocampal tissue, with CREB inhibition abolishing the antidepressant effects, indicating that CREB-mediated signaling plays an essential role in neurogenesis and behavioral improvement [87]. These epigenetic modifications may underlie the durability of therapeutic effects after treatment discontinuation (Fig. 3E).

### Mechanism integration and therapeutic implications

The pathways described above do not operate independently but form an interconnected regulatory network. Neuroinflammation suppresses BDNF expression and impairs synaptic function; conversely, BDNF signaling can attenuate microglial activation [21, 64, 83]. Glutamatergic modulation via NMDA antagonism triggers BDNF release and synaptogenesis, while enhanced monoamine signaling influences inflammatory cytokine production [21, 64]. This interdependence creates opportunities for synergistic therapeutic strategies [21, 25, 88].

Temporal Dynamics of Mechanistic Engagement. Different mechanisms operate on distinct timescales, and effective formulations must address this temporal complexity [25, 28]. Rapid-acting agents (ketamine) modulate synaptic transmission within minutes, providing acute relief [86]. Anti-inflammatory effects typically require hours to days for cytokine normalization [21, 25]. Neuroplasticity enhancement-involving gene expression, protein synthesis, and structural remodeling—unfolds over days to weeks [21, 25]. Intranasal formulations can be engineered to match these temporal requirements: in situ gelling systems provide sustained release aligning with neuroplasticity kinetics, while micellar formulations enable rapid brain exposure for acute intervention [25.28]. This temporal orchestration represents a sophisticated therapeutic strategy uniquely enabled by intranasal delivery [28, 63].

Comparative Efficacy Across Depression Subtypes. The relative importance of each mechanism may vary by depression subtype [21, 89]. Treatment-resistant depression, often associated with prominent neuroinflammation and glutamatergic dysfunction, may preferentially benefit from agents targeting these pathways [21, 89]. Clinical trials of esketamine nasal spray in treatment-resistant depression have demonstrated significant and clinically meaningful reductions in MADRS scores vs. placebo at day 28, with effect sizes of 0.48–0.63, and significant improvement observed as early as 24 h post-first dose [86]. Anhedonia-predominant phenotypes might respond better to therapies enhancing dopaminergic transmission and reward circuit function [21]. The flexibility of intranasal carriers-allowing surface modification for cell-type-specific targeting and controlled release for pathway-appropriate kinetics-positions this platform for personalized psychiatric interventions [25, 88].

Behavioral Manifestations of Mechanistic Convergence. The ultimate validation of mechanistic engagement is improvement in depression-like behaviors [21, 64]. Intranasal materials consistently demonstrate efficacy across multiple behavioral domains: (1) Despair-like behavior (forced swim test, tail suspension test): Reduced immobility time following BDNF, ketamine, venlafaxine nanoparticles, or other active agent administration [28, 64]. (2) Anhedonia (sucrose preference test): Increased sucrose consumption with BDNF, plant-derived extracellular vesicles, or other bioactive compound delivery [25, 88]. Studies using Chlorella vulgaris-derived extracellular vesicles in thermosensitive hydrogel (EVs@IN) demonstrated rapid and potent alleviation of depressive- and anxiety-like behaviors in LPS-induced and CUMS mouse models, with mechanisms involving astrocyte phenotypic transformation, reduced neurotoxic complement C3 release, and activation of the Nrf2-Pgc-1α pathway [10]. (3) Anxiety-like behavior (open field test, elevated plus maze): Increased exploration with various intranasal formulations [80, 85]. (4) Social dysfunction (social interaction test): Enhanced social interaction with optimized delivery systems [88].

These behavioral effects show dose-dependence and vary appropriately across different animal models (chronic mild stress, chronic unpredictable stress, social defeat), confirming the translational relevance of preclinical findings [21, 64, 83].

### Translational perspectives

The mechanistic insights gained from preclinical studies inform clinical development in several ways [21, 90]. First, demonstrating that an intranasal formulation not only elevates brain drug levels but also engages specific pathways (increased hippocampal BDNF, reduced microglial activation) strengthens the rationale for its therapeutic use [8, 91, 92]. For example, studies on intranasal sihosogansan have demonstrated rapid antidepressant activity through activation of GABAergic and BDNF/TrkB/ERK pathways, with effects observed within 30 min of administration, providing a strong mechanistic foundation for clinical translation [8]. Second, identifying biomarkers that reflect pathway engagement—such as plasma BDNF, inflammatory cytokines, or neuroimaging markers of circuit connectivity—enables patient stratification and treatment monitoring [5, 83]. Research has shown that microglial stimulation via intranasal lipopolysaccharide produces antidepressant effects through ERK1/2-mediated BDNF synthesis, suggesting that peripheral markers of microglial activity could serve as predictive biomarkers [91]. Third, understanding the temporal profile of mechanistic engagement guides the selection of outcome measures and assessment time points in clinical trials [21, 90]. Pharmacokinetic studies of intranasal formulations, such as escitalopram-loaded chitosan nanoparticle in situ gels, have demonstrated 4.67-fold higher brain Cmax and 13.31-fold higher AUC compared to oral administration, with prolonged mean residence time, informing optimal dosing intervals for clinical study design [90]. Clinical comparisons of intravenous ketamine versus intranasal esketamine in treatment-resistant depression have further validated these temporal considerations, showing that IV ketamine achieves significant response after one treatment while intranasal esketamine requires two treatments, with overall response rates of 49.22% and 39.55% respectively [90].

In summary, intranasal delivery systems ameliorate depression-like behaviors through convergent mechanisms that span molecular, cellular, and circuit levels [91, 92]. By enabling efficient brain targeting of diverse therapeutic agents and allowing precise control over release kinetics [93], these formulations can engage neuroplasticity, neuroinflammation, neurotransmitter systems, and neural circuits in a coordinated manner [8, 91, 92]. This mechanistic integration, rather than any single pathway, underlies the superior efficacy observed in preclinical models and holds promise for advancing depression treatment toward more rapid, sustained, and personalized interventions [21, 91].

## Clinical translation and challenges of intranasal antidepressants

The translation of intranasal drug delivery systems from preclinical research to clinical application has advanced considerably, with esketamine nasal spray representing the first regulatory approval in this class [94–96]. However, the clinical development landscape reveals both opportunities and substantial challenges [95]. This section synthesizes key findings from clinical trials, critically evaluates efficacy and safety patterns, and examines the multifaceted barriers to widespread clinical adoption [97, 98] (Table 3).

**Table 3:** Clinical translation status of intranasal administration for antidepressants: From evidence to challenges

Dimensions	Core Content/Progress	Key Data/Results	Current Challenges and Future Directions
Clinical efficacy evidence	Esketamine nasal spray (Spravato^®^) in TRD patients:	After 4 weeks of treatment, the response rate was 48% vs. placebo 28%; Remission rate 33% vs. 15% [99,100]. In patients with suicidal ideation: MADRS scores significantly decreased within 24 h of administration [100,101].	Challenges: Approximately 30–50% of patients are non-responders, and there is a lack of reliable predictive biomarkers; the relapse rate is high after discontinuation. Direction: Achieve precise patient stratification by combining biomarkers (such as neuroimaging, metabolomics).
	Researching new formulations: Other drugs (such as ketamine and peptides) in nasal nanoformulations.	Preclinical experiments with nano-carriers (such as fluoxetine NLC) showed a 3.2-fold increase in brain concentration and accelerated onset of action [65].	Challenges: Most are in early clinical stages with small sample sizes and a lack of long-term data. Direction: Promote large-scale Phase II/III RCTs to validate efficacy and safety.
Safety evaluation	Local safety (nasal cavity)	Common: transient burning sensation, nasal bleeding (mostly mild). Nasal endoscopy shows overall good mucosal tolerance [102,103].	Challenge: More data is needed on the effects of long-term medication (> 6 months) on olfactory function and mucosal integrity. Direction: Establish a long-term olfactory monitoring and standardized local toxicity assessment system.
	Safety of the whole body and nervous system	Common: Dissociative symptoms, dizziness, nausea (usually transient). Serious adverse reactions (such as respiratory depression) are rare [104,105].	Challenges: Dissociative symptoms affect patient experience and compliance; long-term CNS neurotoxicity risks need to be thoroughly evaluated in non-human primate models. Direction: Optimize dosage and frequency of administration, develop new drugs/carriers with fewer side effects.
Technological bottleneck	Delivery efficiency is uneven	Individual physiological differences in the nasal cavity (mucus thickness, ciliary clearance rate) can lead to differences in drug absorption by several times [4,106].	Directions: Develop individualized dosage prediction models; design smart responsive carriers (pH/enzyme-triggered) to adapt to different environments.
	The production and quality control of preparations	The sterile filtration, lyophilization stability, and batch-to-batch consistency of nano-carriers are the main obstacles to large-scale production [65.^107^].	Direction: Apply QbD (Quality by Design) principles and microfluidic continuous production technology to achieve stable and uniform GMP production.
	Next-generation carrier technologies	Intelligent responsive carriers and multi-drug co-delivery/drug-gene co-delivery systems, showing the potential for synergistic enhancement in preclinical studies [65,108].	Transitioning from “passive delivery” to “active regulation” enables precise spatiotemporal drug release and synergistic treatment through multiple mechanisms.
Future breakthrough direction	Multimodal combined therapy	Preclinical/early clinical studies show that intranasal administration combined with rTMS, psychotherapy, etc., can improve response rates and prolong efficacy. [109,110]	Break through the limitations of single therapies and construct an integrated treatment paradigm of “physical delivery + neuroregulation + behavioral intervention.”
	Policy and Accessibility	Currently, treatment costs are high, insurance coverage is insufficient, and clinicians’ awareness and training need to be popularized. [111,112]	Promote the accumulation of real-world evidence to support insurance decision-making; develop clinical guidelines and conduct physician education to accelerate technology dissemination.

### Clinical trial design considerations

Clinical trials of intranasal antidepressants must balance rigorous efficacy evaluation with the unique considerations of nasal delivery [95, 113]. The design framework across registered studies reveals several consistent elements [94, 113, 114].

Patient Selection Criteria. Trials typically enroll patients with moderate-to-severe depression, defined by Hamilton Depression Rating Scale scores ≥ 20 or Montgomery-Åsberg Depression Rating Scale (MADRS) scores ≥ 28 [94, 95, 98]. For treatment-resistant depression (TRD) studies, inclusion requires non-response to at least two oral antidepressants of different mechanisms in the current episode, as assessed using standardized tools such as the Massachusetts General Hospital Antidepressant Treatment Response Questionnaire (MGH-ATRQ) [95, 98]. Critically, nasal delivery introduces specific exclusion criteria related to nasal anatomy and function: chronic sinusitis, allergic rhinitis, clinically significant nasal septal deviation, nasal polyps, history of nasal surgery, and olfactory dysfunction—all conditions that may impair drug absorption via olfactory or trigeminal pathways [98, 115, 116].

Control Group Strategies. Three primary control models are employed: placebo nasal spray (typically a solution with bittering agent added to simulate taste) to establish absolute efficacy [95, 118]; oral standard-of-care antidepressants (e.g., sertraline, escitalopram, duloxetine, venlafaxine XR) to evaluate relative efficacy [114, 119]; and active comparator arms (e.g., extended-release quetiapine or intravenous ketamine) to assess non-inferiority [97, 114, 119]. Most contemporary trials combine an oral antidepressant background therapy with either active or placebo nasal spray, ensuring all patients receive standard treatment while isolating the incremental benefit of intranasal delivery [95, 98, 113].

Dosing Regimens and Procedures. Trials typically comprise a 4-week induction phase (dosing twice weekly) followed by a maintenance phase of 6–12 months (weekly, biweekly, or individualized dosing based on symptom severity) [94, 98, 119]. For treatment-resistant depression, approved doses range from 56 mg to 84 mg, with elderly patients (≥ 65 years) initiating at 28 mg [98, 119]. For major depressive disorder with acute suicidal ideation, 84 mg twice weekly for 4 weeks is recommended [98]. Administration procedures are standardized to minimize variability: head tilt at 30–45°, specified number of sprays per nostril, avoidance of food for at least 2 h and liquids for 30 min prior, and maintenance of position for 5–10 min post-administration [98]. Nasal corticosteroids or decongestants, if used, should be administered at least 1 h before esketamine [98].

Efficacy and Safety Endpoints. The primary efficacy endpoint is typically change in MADRS score from baseline, with particular attention to rapid onset (within 24 h to 1 week)—the hypothesized advantage of intranasal delivery [94, 95, 118]. The clinically meaningful threshold is often benchmarked at a 6.5-point difference based on pivotal trial designs [95, 118]. Secondary endpoints include response rates (≥ 50% improvement), remission rates (MADRS ≤ 12), relapse prevention (hazard ratios), suicidal ideation measures (C-SSRS), functional outcomes (Sheehan Disability Scale), and quality of life assessments [94, 95, 118, 119]. Safety evaluation encompasses both local effects (nasal irritation, epistaxis, olfactory function) and systemic effects (dissociative symptoms, sedation, blood pressure changes, cognitive function) [95, 97, 98, 118]. Blood pressure monitoring is mandated before administration, at approximately 40 min post-dose (corresponding to peak plasma concentration), and subsequently as clinically warranted [86]. Patients must be observed in a healthcare facility for at least 2 h post-administration until clinically stable [98].

### Clinical evidence: efficacy and safety patterns

Esketamine: The Established Paradigm. Esketamine nasal spray, approved for TRD, provides the most robust clinical dataset [18, 118]. In short-term trials (TRANSFORM studies), esketamine combined with an oral antidepressant achieved MADRS reductions significantly greater than placebo plus antidepressant, with one meta-analysis confirming significantly higher remission rates compared to placebo (RR = 1.371, 95% CI: 1.194 to 1.574, *p* < 0.0001) [18]. Response rates and remission rates consistently favored esketamine across studies [18, 120]. The onset advantage is evident within 24 h—a clinically meaningful differentiation from oral antidepressants, with meta-analyses demonstrating a pooled effect size for intranasal ketamine/esketamine at 24 h of g = 1.247 [121].

For patients with acute suicidal ideation (ASPIRE studies), esketamine demonstrated significant MADRS reduction at 24 h post-dose, with particular benefit in those with a history of suicide attempts [122, 123]. Pooled analysis of ASPIRE I and II showed that treatment with esketamine plus standard of care resulted in significantly shorter time to remission (median 15 vs. 23 days, *p* = 0.005) and consistent remission (23 vs. 50 days, *p* = 0.007) compared to placebo plus standard of care [123]. Long-term maintenance data (SUSTAIN studies) show sustained efficacy, with improvement in depression generally persisting among participants who remained on maintenance treatment [118]. The SUSTAIN-3 long-term extension study, with total exposure of 3777 cumulative patient-years and mean exposure of 42.9 months, demonstrated that mean MADRS total score decreased during induction and this reduction persisted during optimization/maintenance, with 49.6% of participants in remission at the maintenance phase endpoint [118]. Real-world studies corroborate these findings, with analysis of almost 5 years of real-world use (1,486,213 outpatient treatment sessions completed by 58,483 patients) demonstrating consistency with the established safety profile from clinical studies [124].

Emerging Formulations. Clinical investigation of intranasal formulations for conventional antidepressants remains at earlier stages. Phase I/II studies consistently demonstrate enhanced brain bioavailability and faster brain penetration compared to oral administration. Small proof-of-concept studies suggest potential for accelerated onset and improved tolerability, but adequately powered randomized controlled trials are lacking [121].

Racemic ketamine nasal spray has shown promise in clinical studies. Meta-analyses have evaluated the effect of intravenous, intranasal, and oral ketamine in mood disorders, with intranasal ketamine demonstrating pooled effect sizes of g = 1.247 at 24 h and g = 1.018 at 7–20 days [121]. However, controlled data are limited, and regulatory development has focused on the esketamine enantiomer [18, 121].

Effect Size and Subgroup Patterns. Meta-analyses yield significant effect sizes for intranasal antidepressants in TRD, with dose-dependent effects [18]. A 2024 meta-analysis confirmed that intranasal esketamine exhibited significantly higher remission rates compared to placebo (RR = 1.371, 95% CI: 1.194 to 1.574, *p* < 0.0001), with subgroup analysis revealing that the 84 mg and flexible doses were particularly effective [18]. Comparative effectiveness research has shown that both rTMS (β: −5.35 [95% CI, −8.77, −1.93]) and intranasal esketamine (−2.89 [−5.38, −0.40]) were superior at reducing depression severity when compared with initiating a new antidepressant medication [125]. Subgroup analyses suggest enhanced efficacy in difficult-to-treat populations, with consistent benefits observed across various patient subgroups [120, 125, 126].

Safety Profile. Adverse effects are predominantly mild-to-moderate and transient [18, 118, 124]. Long-term safety data from SUSTAIN-3 (1148 patients enrolled, 3777 cumulative patient-years) identified the most common adverse events as headache (36.9%), dizziness (33.9%), nausea (33.6%), dissociation (25.5%), nasopharyngitis (23.8%), somnolence (23.1%), dysgeusia (20.2%), and back pain (20.0%) [118]. Real-world analysis of 1,486,213 treatment sessions found sedation, dissociation, and increased blood pressure reported in 34.7%, 41.0%, and 0.9% of sessions, respectively, with serious adverse events reported in < 0.1% of treatment sessions [124]. Systemic effects are short-lived (mostly < 2 h) and dose-dependent [118, 124]. Long-term data show no evidence of dependence or abuse with monitored, intermittent dosing [118, 124, 127]. A comprehensive review confirmed that long-term clinical studies have not documented instances of abuse, misuse, addiction, or withdrawal, with no register of illicit acquisition of esketamine or its tampering for obtaining ketamine found in the literature [127]. Serious adverse events are rare, and analysis of almost 5 years of real-world use identified no new safety signals [124].

### Critical analysis: limitations and challenges

Despite encouraging efficacy signals, several limitations temper enthusiasm and highlight gaps between clinical trials and real-world applicability [95, 128] (Table 3).

Generalizability Concerns. Trial populations are highly selected, typically excluding patients with significant medical comorbidity, substance use disorders, or acute suicide risk—precisely the populations where novel treatments are often needed most [95, 128]. A 2025 individual patient data meta-analysis re-evaluating 7 RCTs including 1505 patients noted that most patients displayed low-stage treatment resistance, limiting generalizability to more refractory populations [2, 17]. Real-world analysis of 833 patients initiating esketamine therapy found considerable complexity: 60.2% had at least one prescription fill for a drug with known esketamine interaction, 20% had prescription fills for ≥ 11 drugs, and 18.6% had ≥ 7 drug-prescribing providers [129, 130]. Whether efficacy extends to broader, more complex populations remains uncertain [95, 128].

Trial Design Heterogeneity. Substantial variability across studies complicates cross-trial comparisons and meta-analytic synthesis [83, 95]. Key differences include control conditions (placebo spray vs. active comparator), outcome measures, treatment duration, dosing regimens, and patient populations [18, 95]. A 2024 meta-analysis of 9 studies comprising 1752 patients found that while intranasal esketamine exhibited significantly higher remission rates (RR = 1.371, 95% CI: 1.194–1.574, *p* < 0.0001), heterogeneity across trials limited definitive conclusions about optimal protocols [95]. An IPD meta-analysis confirmed that the clinical relevance of the benefit remains unclear, with a mean MADRS difference of only −2.94 points (95% CI: −5.39 to −0.48) at 4 weeks—below the 6.5-point threshold considered clinically significant in pivotal trial design [95].

Individual Variability in Delivery Efficiency. Nasal drug absorption varies significantly with physiological factors [2, 131]. A comprehensive review highlighted that the nasal cavity comprises distinct anatomical regions with varying permeability, and factors such as mucus thickness (normally 10–100 μm, up to 500 μm in rhinitis), mucociliary clearance rate (5–10 mm/min normally, 2–3 mm/min in smokers), and enzymatic degradation introduce substantial inter-individual variability [2, 131]. These factors-including allergic rhinitis, septal deviation, and nasal pathology-can significantly impair drug absorption via olfactory or trigeminal pathways but remain unaddressed in current dosing strategies [2, 131].

Brain Targeting Efficiency. Current intranasal formulations achieve variable brain targeting efficiencies [25, 132]. Preclinical studies demonstrate that optimized formulations can achieve Drug Targeting Efficiency (DTE) values exceeding 800% and Direct Transport Percentage (DTP) over 90% in animal models [108]. For example, paliperidone-loaded thermosensitive in situ gel achieved brain Cmax 2.66-fold higher than IV administration, with DTE of 807% and DTP of 91% [108]. Pharmacokinetic modeling of remoxipride after intranasal administration revealed total bioavailability of 89%, of which 75% was attributed to direct nose-to-brain transport [126]. However, these optimized preclinical results do not always translate to human applications, and significant room for improvement remains [2, 131].

Real-World Feasibility Barriers. Practical requirements of intranasal antidepressant therapy pose implementation challenges [128–132]. Esketamine administration requires in-clinic dosing under supervision, 2-h post-dose monitoring, and twice-weekly visits initially—a burden that limits access [128]. Especially, the most patients generally required more time than label recommendation to complete ESK induction phase, and most went on to have 12 or more ESK sessions [129, 130]. An implementation science assessment using the Consolidated Framework for Implementation Research (CFIR) revealed that despite established efficacy and effectiveness, most psychiatric treatment settings do not offer intranasal esketamine, with limited integration into mainstream psychiatric treatment [128] (Table 3).

Regulatory and Reimbursement Hurdles. Regulatory pathways for novel intranasal formulations remain complex [95.123]. A commentary on ketamine as a new treatment for severe depression highlighted that Spravato^®^ is priced at $600–$900/dose compared to ~$5/dose for generic ketamine, with an ~ AUD$100 million annual government investment requested in Australia rejected twice, leaving this treatment largely inaccessible [123]. Reimbursement gaps persist globally, and bioequivalence standards differ between regions, forcing additional trials for global development [95, 123].

Clinical Awareness and Training. Implementation research indicates limited clinician familiarity and training [128]. The CFIR analysis identified that only a minority of eligible TRD patients in the US have received intranasal therapy, with barriers including inadequate clinician knowledge of rapid-onset mechanisms, limited training in nasal delivery techniques, and systemic factors limiting adoption [128].

### Future directions

Addressing the challenges will require coordinated advances across multiple domains:

Advanced Delivery Technologies. Next-generation carriers aim to improve brain targeting efficiency. Surface modification with cell-penetrating peptides (Tat, RVG) can significantly increase brain concentrations [133, 134]. Studies have demonstrated that Tat peptide-modified polymer micelles (MPEG-PCL-Tat) achieve significantly higher brain concentrations compared to unmodified micelles, with the Tat peptide enabling enhanced penetration across the nasal mucosa and target cell membranes [133, 134]. The optimal particle size for nose-to-brain delivery is approximately 100 nm, which facilitates transport through endocytic pathways of sustentacular or neuronal cells in the olfactory epithelium [134]. Extracellular vesicles (exosomes) leverage natural transport mechanisms, achieving efficient brain targeting after intranasal delivery [135–137]. Research has shown that exosome-sheathed reactive oxygen species-responsive nanogels loaded with PACAP and estrogen demonstrate efficient cellular uptake and blood-brain barrier penetration, producing rapid-onset antidepressant effects in ovariectomized mice under chronic unpredictable mild stress [135]. Adipose mesenchymal stem cell-derived exosomes administered intranasally ameliorate depressive-like behaviors by inhibiting NLRP3 inflammasome activation, reducing pro-inflammatory cytokine release, and regulating AMPK/mTOR-mediated autophagy [136]. Stem cell-derived exosomes have emerged as promising therapeutic tools for central nervous system diseases due to their ability to deliver bioactive molecules across the blood-brain barrier [137].

Multimodal Treatment Integration. Combining intranasal delivery with other modalities may enhance outcomes [110, 138, 139]. Repetitive transcranial magnetic stimulation (rTMS) has been positioned as a key intervention in sequential treatment algorithms for treatment-resistant depression, with comparative effectiveness studies demonstrating that rTMS significantly outperforms medication switch and achieves numerically greater response rates compared to antipsychotic augmentation [138]. Preliminary studies suggest synergy between intranasal interventions and psychotherapy; a randomized controlled trial is currently evaluating whether intranasal oxytocin augmentation enhances the therapeutic effect of cognitive-behavioral therapy for major depressive disorder [110]. Combinations with vagus nerve stimulation are under investigation, as vagus nerve stimulation represents a novel neuromodulation approach that may complement intranasal delivery strategies [139].

Precision Medicine Approaches. Biomarker-guided patient selection could improve response rates [95, 140]. Metabolomic profiling and neuroimaging approaches are being developed to identify patients most likely to benefit from specific intranasal regimens [95]. Emerging research suggests that gut microbiota composition correlates with treatment outcomes; intranasal delivery of Bifidobacterium encapsulated in mesoporous silica nanoparticles has been shown to reach the gastrointestinal tract via the brain-gut axis, modulating intestinal microbiota and reducing neuroinflammation [140]. These findings suggest opportunities for microbiome modulation as a therapeutic strategy [140]. Regulatory pathways for biomarker-driven approaches are evolving, with increasing emphasis on patient stratification based on underlying pathophysiology [95].

Real-World Evidence Generation. Large-scale observational studies are evaluating intranasal antidepressants in diverse populations, including elderly patients and those with comorbid medical illness [95]. Real-world data will inform optimal dosing, identify risk factors, and support cost-effectiveness analyses [95]. Regulatory acceptance of real-world evidence is expanding; the FDA’s Real-World Evidence Program provides pathways for utilizing real-world data to support regulatory decisions, which may accelerate approval pathways for novel intranasal formulations [95]. Comparative effectiveness research frameworks are essential for positioning intranasal therapies within sequential treatment algorithms, addressing the critical question of which intervention to choose and when in the clinical course of treatment-resistant depression [95, 128].

In summary, intranasal antidepressants have established clinical proof-of-concept, particularly for treatment-resistant depression and rapid symptom relief. However, realizing their full potential requires overcoming substantial barriers in delivery efficiency, practical feasibility, and healthcare system integration. Advances in delivery technology, precision medicine, and real-world evidence generation-coupled with regulatory and reimbursement alignment-will determine whether intranasal delivery becomes a mainstream depression treatment or remains a niche intervention for selected populations.

## Safety considerations for intranasal drug delivery systems

The safety profile of intranasal antidepressants is shaped by both the pharmacological agent and the delivery system itself. This section synthesizes available safety data into a structured risk framework, highlighting established findings and critical gaps in long-term human data.

### A structured safety framework

Safety considerations for intranasal delivery systems can be organized into four interconnected domains, each with distinct implications for clinical use and regulatory assessment.

Local Nasal Tolerance. The nasal mucosa is the first interface between formulation and patient. Across clinical studies, mild-to-moderate nasal irritation affects a proportion of patients, manifesting as burning sensation, epistaxis, nasal congestion, and transient hyposmia [141]. In four multicenter, randomized, double-blind, phase III studies evaluating esketamine nasal spray in treatment-resistant depression (TRD), objective evaluation of nasal function using the University of Pennsylvania Smell Identification Test (UPSIT^®^) and the Snap & Sniff^®^ Odor Detection Threshold Test (S&S-T) showed no evidence of adverse impact following esketamine administration [141–143]. Repeated intermittent administration over short-term (4 weeks) or long-term (16–100 weeks) periods had no meaningful impact on assessments of nasal function, and no dose–response relationship was observed between esketamine doses and olfactory test scores [141]. These effects typically diminish with continued treatment and are rarely treatment-limiting [141–143].

However, preclinical data reveal material-specific effects. For PLGA-based formulations, histopathological examination of nasal tissues has shown no lesions of pathological significance, indicating good local tolerability [144]. On the other hand, studies on chitosan nanoparticles have demonstrated that while biocompatible, they can induce immune activation—intranasal immunization with chitosan nanoparticles triggered IL-6 signaling pathway activation and cellular movement of immune cells within nasal-associated lymphoid tissue (NALT) [145, 146]. Water-soluble chitosan (WSC) has been shown to polarize cytokine balance towards Th1 cytokines and modulate inflammatory responses, suggesting potential immunomodulatory effects that require careful evaluation [146]. Long-term effects on olfactory function remain inadequately studied; most clinical trials assess olfaction only through subjective reports rather than quantitative testing, although the esketamine trials represent an exception in employing validated objective measures [141].

Systemic Exposure and Immunogenicity. Following nasal absorption, materials enter systemic circulation either directly through the vascularized nasal mucosa or after drainage from the CNS [15, 147–150]. For nanocarriers, surface properties determine immune interactions. PLGA nanoparticles are biodegradable and biocompatible, with FDA approval for drug administration, and degrade to biocompatible monomers (lactic and glycolic acid) [144, 150]. Studies have demonstrated that intranasal administration of PLGA nanoparticles results in good tolerability without significant local toxicity [144, 150].

Polyethylene glycol (PEG) modification, commonly used to evade immune clearance, may induce anti-PEG antibodies with repeated administration—a consideration for chronic treatment regimens. Lipid-based carriers generally exhibit low immunogenicity, as demonstrated by studies on dextran microspheres and thermogelling ethyl (hydroxyethyl)cellulose (EHEC) systems, which showed no adjuvant effect and did not generate specific IgA or IgG antibodies following intranasal administration [151, 152]. However, high-dose or frequent administration of synthetic polymers could transiently alter local pH or osmolarity. The clinical significance of these effects with intermittent, low-volume intranasal dosing requires further study [15, 153–156].

CNS-Specific Risks. Brain-targeted delivery raises concerns about unintended CNS effects [21, 157–159]. Dissociation and sedation, observed with esketamine, represent on-target pharmacological effects [160–162]. For nanocarriers, concerns include potential neuroinflammation from chronic carrier accumulation, disruption of neuronal function by cationic materials, and unknown effects of long-term carrier residence in brain tissue [21]. Preclinical studies indicate that microglia, the resident immune cells of the CNS, play a critical role in clearing nanoparticles and cellular debris in the olfactory bulb, with sensory experience influencing microglial phagocytic activity [158]. However, materials commonly used in clinical-stage formulations (chitosan, PLGA, phospholipids) have favorable safety profiles. Chitosan-modified PLGA nanoparticles have demonstrated excellent biocompatibility with no mortality, hematological changes, or histopathological alterations in animal studies [163, 164]. Biomineralized silk fibroin nanoparticles administered intranasally for depression treatment have shown no toxicity in major organs or blood indicators, with good safety profiles confirmed by in vivo evaluation [29]. The small doses delivered intranasally and intermittent dosing schedules mitigate many theoretical risks, but dedicated neurotoxicity studies with chronic, clinically relevant dosing regimens are lacking [21].

Long-Term Accumulation and Clearance. The fate of nanocarrier components after brain delivery is a critical knowledge gap [21]. Particles < 100 nm may be internalized by neurons and transported intracellularly, potentially persisting in brain tissue [165–168]. Studies using metal-organic framework (MOF-74-Mg) nanocarriers for intranasal delivery demonstrated that nanoparticles are taken up by 79–85% of neurons and 93–97% of microglia in various brain regions, including the hippocampus and striatum, within 45 min of administration, highlighting the extensive cellular uptake of nanocarriers in the CNS [168]. Clearance mechanisms include degradation by local enzymes, phagocytosis by microglia, and slow diffusion into perivascular spaces [169]. Synthetic polymers like PLGA degrade over weeks to months via hydrolysis to biocompatible monomers (lactic and glycolic acid); lipid components are metabolized by endogenous pathways [170–172]. In vivo safety evaluation of donepezil-loaded chitosan nanosuspension administered intranasally showed no mortality, hematological changes, body weight variations, or histopathological alterations, confirming the favorable safety profile of chitosan-based nanocarriers [173]. However, quantitative data on brain residence time, clearance kinetics, and cellular localization after repeated intranasal dosing in humans are absent. Chronic accumulation studies in appropriate animal models, with dosing regimens mirroring clinical protocols, are needed.

### Clinical safety data: established patterns and gaps

Esketamine: The Reference Case. Esketamine nasal spray provides the most comprehensive clinical safety dataset [95, 159–162, 174, 175]. The safety profile is well-characterized across multiple phase III randomized controlled trials and large-scale real-world analyses. A 2025 systematic review and meta-analysis of 17 RCTs comprising 10,073 patients confirmed that esketamine significantly improves treatment response and remission rates, with safety analysis revealing increased risk of dissociation (RR = 1.98; 95% CI: 1.68–2.28) and hypertension (RR = 1.42; 95% CI: 1.04–1.80), while non-significant elevations were observed for sedation (RR = 1.23; 95% CI: 0.80–1.66) and nausea (RR = 1.10; 95% CI: 0.82–1.37) [175]. An individual patient data meta-analysis of 7 RCTs including 1505 patients found that esketamine increased sedation (RR = 3.70 [2.02–6.78]), dissociation (RR = 2.36 [2.10–2.65]), and overall adverse events (IRR = 3.91 [2.37–6.45]), with no increase in serious adverse events (IRR = 1.35 [0.54–3.40]). These adverse effects are typically transient, with dissociative symptoms resolving within 2 h [95].

A comprehensive five-year real-world safety analysis of 1,486,213 outpatient treatment sessions completed by 58,483 patients in the United States confirmed the consistency of the real-world safety profile with clinical trial findings [124]. Solicited reports of sedation, dissociation, and increased blood pressure were received for 34.7%, 41.0%, and 0.9% of treatment sessions, respectively, with serious adverse events reported in less than 0.1% of treatment sessions [124]. The Risk Evaluation and Mitigation Strategy (REMS) program—requiring supervised administration under healthcare professional supervision, use of two or three devices per dose with 5-min rest intervals between devices, and a minimum 2-h post-dose monitoring period—has effectively mitigated risks in real-world settings [22, 124]. No overdose cases were reported in the clinical trial program including 2,283 subjects, and the maximum tested dose of 112 mg demonstrated dose-dependent increases in adverse events such as dizziness, nasal congestion, and dissociative symptoms [124].

Investigational Formulations. Safety data for novel nanocarrier formulations derive primarily from preclinical studies and early-phase clinical investigations [164, 172]. For chitosan-modified PLGA nanoparticles, comprehensive in vitro and in vivo safety evaluations have demonstrated favorable profiles. Cellular assays on mammalian cells showed cell viability > 60% even at maximum concentrations of paroxetine-loaded chitosan-PLGA nanoparticles (PAR-CS-PLGA-NPs), with significantly higher cellular uptake than unmodified PLGA-NPs [164]. Histopathological studies on nasal epithelium confirmed no damage or inflammation when treated with PAR-CS-PLGA-NPs [164]. Similarly, quetiapine-loaded poloxamer-chitosan-PLGA nanoparticles (QF-PLGA-ISG) demonstrated safe and effective uptake and permeation against RPMI-2650 cell lines and MatTek EpiNasal^™^ 3D tissue models, with ex-vivo hemolysis studies confirming no hemolysis compared to negative control [172]. Histological reports further confirmed the safety of optimized formulations [172]. For investigational esketamine formulations, studies conducted in Chinese and US populations with TRD (*n* = 252) reported no new safety signals, with adverse events consistent with the established safety profile [162]. However, these studies remain limited in size (typically 20–60 patients for novel carrier formulations) and duration (weeks to months), and often exclude patients with nasal pathology, limiting generalizability to broader populations.

## Outlook and future directions

**Fig. 4:**
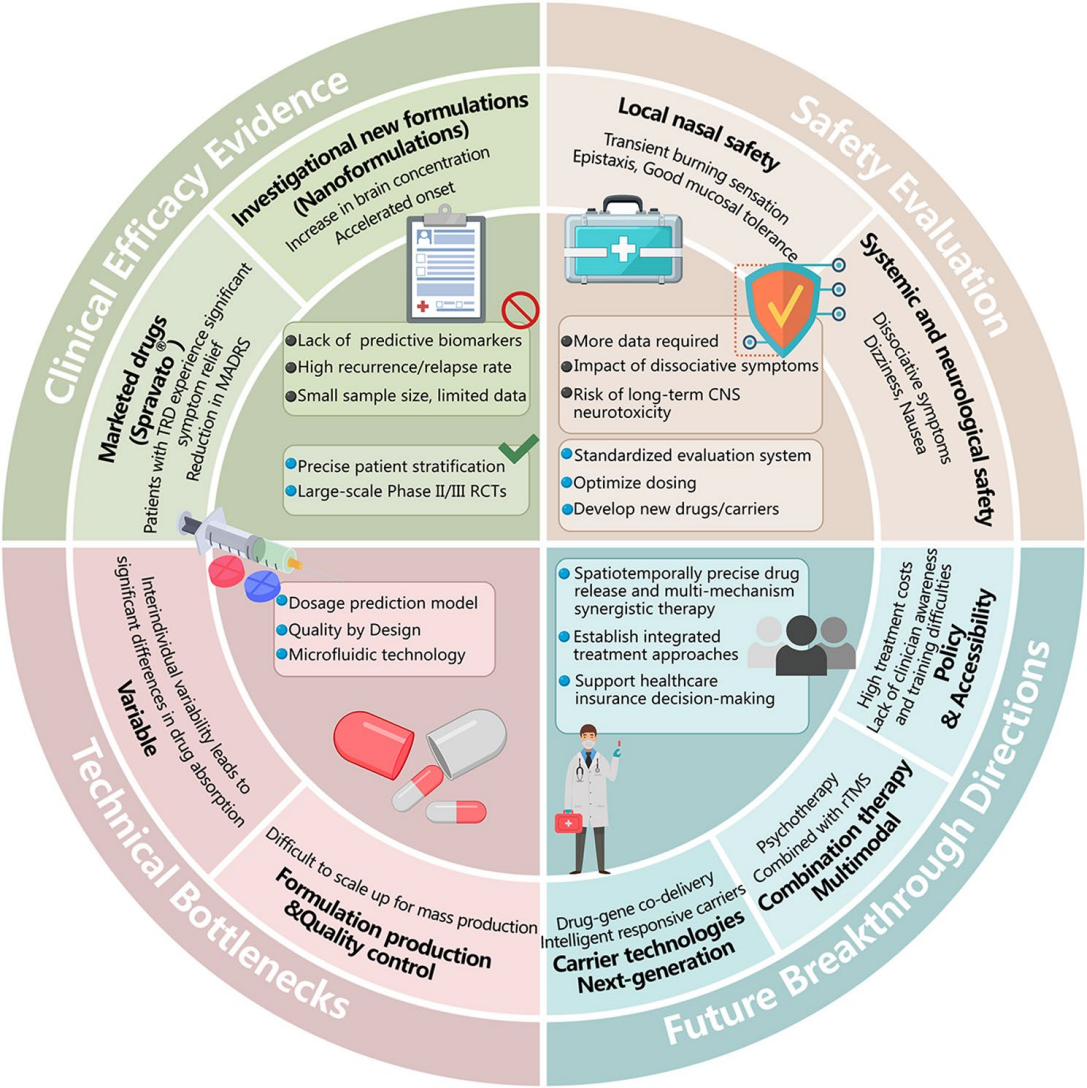
Multidimensional synergistic mechanisms of antidepressant intranasal drug delivery. This schematic integrates the current evidence base, ongoing challenges, and future directions. It highlights promising applications across precision medicine and nanomedicine, alongside clinical hurdles such as variable efficacy and safety concerns. The path forward is outlined through key strategies: Stratifying patients for targeted trials, developing personalized dosing models, and optimizing safety profiles. The future vision emphasizes advancing precision therapies, establishing multimodal treatments, and generating real-world evidence to inform policy and clinical adoption

The field of intranasal antidepressant delivery stands at a critical juncture, with established clinical proof-of-concept for esketamine and a pipeline of advanced formulations approaching clinical evaluation. This section outlines evidence-grounded future directions while acknowledging uncertainties inherent in forecasting technological and clinical trajectories (Fig. 4).

### Current landscape: strengths, weaknesses, opportunities, and threats

Strengths. Intranasal delivery offers fundamental advantages: bypass of the blood-brain barrier, rapid brain penetration (minutes to hours vs. weeks for oral antidepressants), reduced systemic exposure, and suitability for patients unable to take oral medications [177, 178]. The platform is versatile, applicable to diverse therapeutic agents (small molecules, peptides, proteins, nucleic acids) [179]. Esketamine’s regulatory approval demonstrates clinical and regulatory feasibility [13, 18].

Weaknesses. Brain targeting efficiency remains modest (5–15%), limiting dose precision [5, 15]. Individual anatomical and physiological variability introduces unpredictable drug exposure [14]. Formulation complexity increases manufacturing costs [179]. Clinical adoption is constrained by administration burden (in-clinic dosing, monitoring) and limited prescriber familiarity [13, 17]. The evidence base for novel formulations beyond esketamine remains thin [11].

Opportunities. Advances in nanocarrier engineering could substantially improve brain targeting efficiency (target: 25–30%) [180, 181]. Stimuli-responsive materials enable region-specific or pathology-triggered release [183]. Biomarker-guided patient selection could double response rates [184]. Multimodal combinations with psychotherapy, neuromodulation) may enhance and sustain efficacy [20]. Real-world evidence generation can fill evidence gaps and support regulatory decisions [185].

Threats. Regulatory pathways for reformulated drugs remain uncertain; demonstration of superiority to oral versions may be required [186]. Reimbursement decisions may limit access if cost-effectiveness is not convincingly demonstrated. Competition from other rapid-acting antidepressants could erode the intranasal niche. Manufacturing scale-up challenges could delay commercialization. Safety concerns emerging from long-term use could restrict indications.

### Development scenarios and timelines

Rather than assigning speculative numerical scores or fixed approval dates, we describe development trajectories based on technical complexity and regulatory precedent:

Immediate-Term. Esketamine-like formulations represent the current standard [19, 187]. Near-term advances will focus on optimizing dosing regimens, expanding indications (bipolar depression, anxiety disorders), and generating real-world evidence in diverse populations [13, 17]. Reformulation of existing antidepressants using established nanocarriers (PLGA nanoparticles, lipid-based carriers) will advance through phase II/III trials, with regulatory approval contingent on demonstrating meaningful clinical advantages over oral versions [188, 189]. Device improvements (dose counters, ergonomic designs) will enhance usability [14].

Medium-Term. Second-generation nanocarriers with active targeting (ligand-modified nanoparticles) and controlled release (stimuli-responsive materials) will enter clinical evaluation [180, 190]. These systems aim to achieve brain targeting efficiencies of 20–30% and enable weekly or monthly dosing [5, 181]. Co-delivery platforms combining agents with synergistic mechanisms (rapid-acting glutamate modulator + sustained-release neurotrophic factor) will be tested in proof-of-concept trials [98, 191]. Biomarker-guided patient selection will move from exploratory to interventional studies [192, 193].

Long-Term. Widespread clinical integration will depend on cumulative evidence from multiple sources: randomized trials in representative populations, long-term safety registries, cost-effectiveness analyses, and implementation research [186, 194]. Precision medicine approaches—integrating genomics, metabolomics, neuroimaging—could enable individualized formulation selection and dose titration [195, 196]. However, the pace and extent of adoption will be shaped by healthcare system factors (reimbursement policies, prescriber training, patient access) as much as by technological advances [197, 198].

### Critical research priorities

To realize the potential of intranasal antidepressants, the field must prioritize several interconnected research directions:

Optimizing Brain Targeting. Improving delivery efficiency from current 5–15% to 20–30% would enhance dose precision and reduce variability. Priority approaches include systematic evaluation of targeting ligands, engineering of mucus-penetrating coatings, and development of stimuli-responsive systems. Comparative studies directly measuring brain concentrations (via PET or CSF sampling) in humans are needed to validate preclinical findings [178, 199].

Establishing Long-Term Safety. The most critical evidence gap is long-term safety data for novel formulations. Priority studies include 12–24 month animal toxicology studies with clinically relevant, intermittent dosing; human studies with quantitative olfactory testing and nasal endoscopy; CNS imaging to assess for accumulation or structural changes; and cognitive function assessments. Registry-based post-marketing surveillance will be essential [95–98].

Defining Optimal Patient Populations. Identifying predictors of response is essential for cost-effective use. Priority research includes harmonized collection of biospecimens and imaging data across trials to enable pooled analyses; validation of candidate biomarkers (tryptophan metabolites, inflammatory markers, default mode network connectivity) in prospective studies; and pragmatic trials comparing biomarker-guided vs. unselected treatment [200, 201].

Demonstrating Comparative Effectiveness. Novel formulations must demonstrate meaningful advantages over existing treatments. Priority comparisons include head-to-head trials against oral antidepressants (including novel rapid-acting agents); evaluations of functional outcomes (work productivity, social function) beyond symptom scores; and cost-effectiveness analyses incorporating healthcare utilization and quality-adjusted life years [202].

Addressing Implementation Barriers. Priority research includes simplified administration protocols (shorter observation periods, home-based dosing for stable patients); telemedicine-supported monitoring to reduce visit burden; and implementation science studies identifying barriers and facilitators in diverse practice settings [17, 203].

### Concluding perspective

Intranasal antidepressant delivery represents a genuine therapeutic advance, particularly for patients with treatment-resistant depression and those requiring rapid symptom relief [204, 205]. The platform’s fundamental advantages—bypassing the blood-brain barrier, achieving rapid brain penetration, reducing systemic exposure—address limitations of conventional pharmacotherapy [17, 206]. Esketamine’s regulatory approval and clinical uptake demonstrate that these theoretical advantages translate to real-world benefit [160, 207].

However, the field stands at an early stage. Most formulations remain investigational; brain targeting efficiency is modest [21, 208]; individual variability is substantial [209]; and long-term safety data are limited [178, 204]. The promise of precision intranasal therapy—with formulations matched to patient characteristics, doses titrated to individual physiology, and combinations tailored to symptom profiles—requires substantial additional evidence [110].

Realizing this vision demands coordinated effort across academia, industry, and regulatory bodies [63]. Academic research should prioritize mechanistic understanding, biomarker validation, and rigorous comparative studies. Industry must invest in scalable manufacturing, user-friendly devices, and evidence generation for regulatory approval [118]. Regulators and payers must develop clear pathways for evaluating novel formulations, balancing innovation incentives with evidence standards.

The trajectory of intranasal antidepressants will ultimately be determined not by technological sophistication alone, but by demonstrated improvements in patient-relevant outcomes, acceptable safety profiles, and practical feasibility in real-world healthcare settings [210, 211]. If these conditions are met, intranasal delivery could become an integral component of depression treatment—not replacing existing therapies, but filling critical gaps in the therapeutic armamentarium [9]. If not, it may remain a niche intervention for selected populations. The next decade of research will determine which scenario unfolds.

## Data Availability

Data sharing is not applicable to this article as no datasets were generated or analyzed during the current study.

## Abbreviations

BBB: Blood-brain barrier
BDNF: Brain-derived neurotrophic factor
CFIR: Consolidated Framework for Implementation Research
CNS: Central nervous system
CUMS: Chronic unpredictable mild stress
DTE: Drug targeting efficiency
DTP: Direct transport percentage
GI: Gastrointestinal
HDAC5: Histone deacetylase 5
HTCC: Hydroxypropyltrimethyl ammonium chloride chitosan
IN: Intranasal
IV: Intravenous
MADRS: Montgomery-Asberg Depression Rating Scale
MGH-ATRQ: Massachusetts General Hospital Antidepressant Treatment Response Questionnaire
MOF: Metal-organic framework
NALT: Nasal-associated lymphoid tissue
NLCs: Nanostructured lipid carriers
PAR-CS-PLGA-NPs: Paroxetine-loaded chitosan-PLGA nanoparticles
PEG: Polyethylene glycol
QF-PLGA-ISG: Quetiapine-loaded poloxamer-chitosan-PLGA nanoparticles
REMS: Risk Evaluation and Mitigation Strategy (
rTMS: Repetitive transcranial magnetic stimulation
S&S-T: Snap & sniff^®^ odor detection threshold test
SNRIs: Serotonin-norepinephrine reuptake inhibitors
SSRIs: Selective serotonin reuptake inhibitors
TCA: Tricyclic antidepressant
TRD: Treatment-resistant depression
UPSIT: University of Pennsylvania Smell Identification Test
WSC: Water-soluble chitosan
